# Crystal structure of 5-amino-5′-chloro-6-(4-chloro­benzo­yl)-8-nitro-2,3-di­hydro-1*H*-spiro­[imidazo[1,2-*a*]pyridine-7,3′-indolin]-2′-one including an unknown solvent mol­ecule

**DOI:** 10.1107/S1600536814017486

**Published:** 2014-08-06

**Authors:** R. A. Nagalakshmi, J. Suresh, S. Sivakumar, R. Ranjith Kumar, P. L. Nilantha Lakshman

**Affiliations:** a Department of Physics, The Madura College, Madurai 625 011, India; b Department of Organic Chemistry, School of Chemistry, Madurai Kamaraj University, Madurai 625 021, India; c Department of Food Science and Technology, University of Ruhuna, Mapalana, Kamburupitiya 81100, Sri Lanka

**Keywords:** crystal structure, spiro, imidazole, pyridine-indoline C—H⋯π inter­actions, hydrogen bonding

## Abstract

The asymmetric unit of the title compound, C_21_H_15_Cl_2_N_5_O_4_, contains two independent mol­ecules (*A* and *B*) having similar conformations. The amine (NH_2_) group forms an intra­molecular hydrogen bond with the benzoyl group, giving an *S*(6) ring motif in both mol­ecules. The central six-membered rings adopt sofa conformations and the imidazole rings are planar (r.m.s deviations = 0.0150 and 0.0166 Å). The pyridine and imidazole rings are inclined to one another by 3.54 (1) and 3.03 (1)° in mol­ecules *A* and *B*, respectively. In the crystal, mol­ecules are linked by N—H⋯O hydrogen bonds, forming chains along the *a* axis which enclose *R*_2_^2^(16) ring motifs. The rings are linked by weak N—H⋯O and C—H⋯O hydrogen bonds and C—H⋯π inter­actions forming sheets lying parallel to (001). A region of disordered electron density, most probably disordered solvent mol­ecules, occupying voids of *ca* 753 Å^3^ for an electron count of 260, was treated using the SQUEEZE routine in *PLATON* [Spek (2009 ▶). *Acta Cryst.* D**65**, 148–155]. Their formula mass and unit-cell characteristics were not taken into account during refinement.

## Related literature   

For a similar structure, a pharmacologically active pyridine-related compound, see: Nagalakshmi *et al.* (2014 ▶).
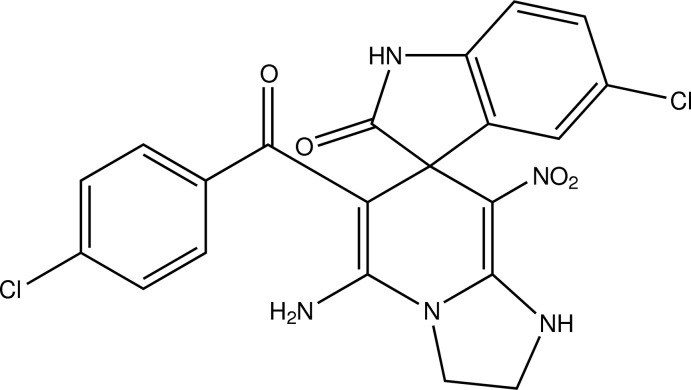


## Experimental   

### Crystal data  

C_21_H_15_Cl_2_N_5_O_4_
*M* *_r_* = 472.28
Triclinic, 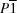

*a* = 12.9805 (7) Å
*b* = 13.2085 (8) Å
*c* = 16.7175 (10) Å
α = 105.713 (2)°
β = 103.367 (2)°
γ = 91.051 (2)°
*V* = 2674.4 (3) Å^3^
*Z* = 4
Mo *K*α radiation
μ = 0.27 mm^−1^
*T* = 293 K
0.21 × 0.19 × 0.18 mm

### Data collection  

Bruker Kappa APEXII diffractometer
Absorption correction: multi-scan (*SADABS*; Bruker, 2004 ▶) *T*_min_ = 0.967, *T*_max_ = 0.974
87748 measured reflections
11499 independent reflections
7063 reflections with *I* > 2σ(*I*)
*R*_int_ = 0.044

### Refinement  

*R*[*F* ^2^ > 2σ(*F* ^2^)] = 0.058
*wR*(*F* ^2^) = 0.181
*S* = 1.11
11499 reflections
577 parameters
2 restraints
H-atom parameters constrained
Δρ_max_ = 0.38 e Å^−3^
Δρ_min_ = −0.31 e Å^−3^

### 

Data collection: *APEX2* (Bruker, 2004 ▶); cell refinement: *SAINT* (Bruker, 2004 ▶); data reduction: *SAINT*; program(s) used to solve structure: *SHELXS97* (Sheldrick, 2008 ▶); program(s) used to refine structure: *SHELXL97* (Sheldrick, 2008 ▶); molecular graphics: *PLATON* (Spek, 2009 ▶); software used to prepare material for publication: *SHELXL97*.

## Supplementary Material

Crystal structure: contains datablock(s) global, I. DOI: 10.1107/S1600536814017486/su2757sup1.cif

Structure factors: contains datablock(s) I. DOI: 10.1107/S1600536814017486/su2757Isup2.hkl

Supporting information file. DOI: 10.1107/S1600536814017486/su2757Isup3.cml

**Fig. 1.**
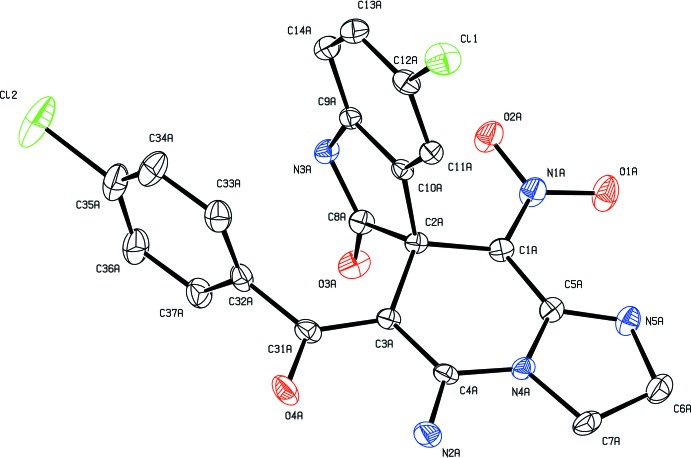
The molecular structure of molecule A of the title compound, with atom labelling. Displacement ellipsoids are drawn at the 20% probability level. H-atoms have been omitted for clarity.

. DOI: 10.1107/S1600536814017486/su2757fig1.tif
The mol­ecular structure of mol­ecule A of the title compound, with atom labelling. Displacement ellipsoids are drawn at the 20% probability level. H-atoms have been omitted for clarity.

**Fig. 2.**
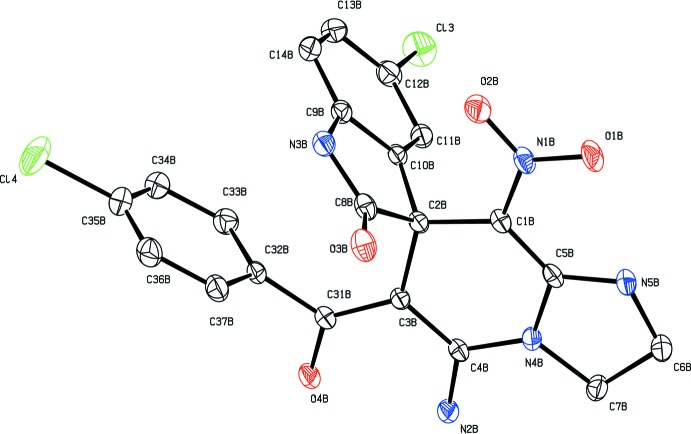
The molecular structure of molecule B of the title compound, with atom labelling. Displacement ellipsoids are drawn at the 20% probability level. H-atoms have been omitted for clarity.

. DOI: 10.1107/S1600536814017486/su2757fig2.tif
The mol­ecular structure of mol­ecule B of the title compound, with atom labelling. Displacement ellipsoids are drawn at the 20% probability level. H-atoms have been omitted for clarity.

**Fig. 3.**
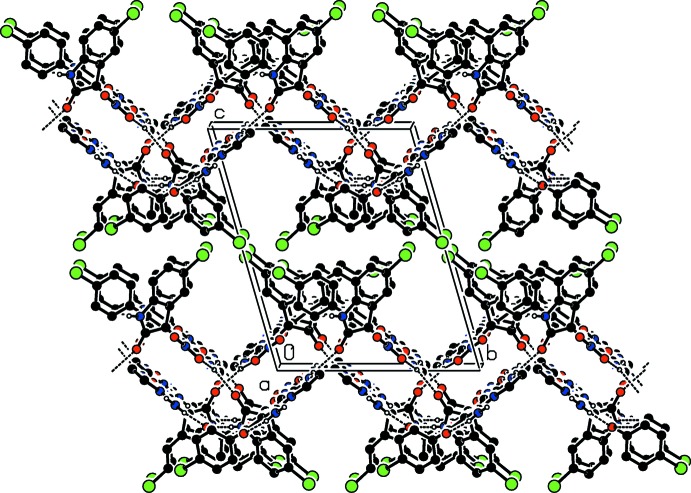
A view along the a axis of the crystal packing of the title compound. Dashed bonds represent N-H···O hydrogen bonds (see Table 1 for details; C bound H atoms have been omitted for clarity).

. DOI: 10.1107/S1600536814017486/su2757fig3.tif
A view along the a axis of the crystal packing of the title compound. Dashed bonds represent N-H⋯O hydrogen bonds (see Table 1 for details; C bound H atoms have been omitted for clarity).

CCDC reference: 1016869

Additional supporting information:  crystallographic information; 3D view; checkCIF report

## Figures and Tables

**Table 1:** Hydrogen-bond geometry (Å, °) *Cg*1 and *Cg*2 are the centroids of rings C1*A*–C5*A*/N4*A* and C1*B*–C5*B*/N4*B*, respectively.

*D*—H⋯*A*	*D*—H	H⋯*A*	*D*⋯*A*	*D*—H⋯*A*
N3*B*—H3*B*⋯O4*A*	0.86	1.91	2.774 (3)	179
N5*A*—H5*A*⋯O1*A*	0.86	2.06	2.592 (3)	119
N5*B*—H5*B*⋯O1*B*	0.86	2.08	2.604 (3)	119
N2*A*—H10⋯O4*A*	0.86	1.85	2.510 (3)	132
N2*B*—H12⋯O4*B*	0.86	1.86	2.515 (3)	132
N3*A*—H3*A*⋯O4*B* ^i^	0.86	1.96	2.823 (2)	177
N5*A*—H5*A*⋯O3*B* ^ii^	0.86	2.42	3.131 (3)	140
N5*B*—H5*B*⋯O3*A* ^iii^	0.86	2.26	2.899 (3)	131
C7*A*—H5⋯O3*A* ^ii^	0.97	2.58	3.302 (3)	132
C7*A*—H6⋯O1*B* ^iv^	0.97	2.59	3.221 (3)	123
C7*A*—H5⋯*Cg*1^ii^	0.97	2.89	3.679 (3)	139
C7*B*—H8⋯*Cg*2^v^	0.97	2.85	3.747 (3)	155

Symmetry codes: (i) 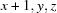; (ii) 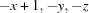; (iii) 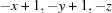; (iv) 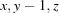; (v) 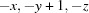.

## supplementary crystallographic information

### S1. Synthesis and crystallization

A mixture of 4-chloro­benzoyl­aceto­nitrile (1.0 mmol), 5-chloro­isatin (1.0 mmol) and 2-(nitro­methyl­ene)imidazolidine were dissolved in 10 ml of EtOH and tri­ethyl­amine (1.0 mmol) was added and the reaction mixture was heated to reflux for 45 min. After completion of the reaction, as evident from TLC, the precipitate was filtered and dried to obtain pure pale brown solid. Colourless crystals of the title compound were obtaind by slow evaporation of a solution in di­methyl sulphoxide (yield 93 %; m.p. 534 K).

### S2. Refinement

H atoms were placed in calculated positions and treated as riding atoms: N—H = 0.86 Å, C—H = 0.93 (aromatic CH), 0.96 (methyl CH_3_) and 0.97 Å (methyl­ene CH_2_) with *U*_iso_(H) = 1.5*U*_eq_(C-methyl) and = 1.2*U*_eq_(N,C) for other H atoms. A region of disordered electron density, most probably disordered solvent molecules, occupying voids of ca. 753 Å^3^ for an electron count of 260, was treated using the SQUEEZE routine in PLATON [Spek (2009). Acta Cryst. D65, 148-155]. Their formula mass and unit-cell characteristics were not taken into account during refinement.

### Crystal data

**Table:**

C_21_H_15_Cl_2_N_5_O_4_	*Z* = 4
*M**_r_* = 472.28	*F*(000) = 968
Triclinic, *P*1	*D*_x_ = 1.173 Mg m^−^^3^
Hall symbol: -P 1	Mo *K*α radiation, λ = 0.71073 Å
*a* = 12.9805 (7) Å	Cell parameters from 2000 reflections
*b* = 13.2085 (8) Å	θ = 2–31°
*c* = 16.7175 (10) Å	µ = 0.27 mm^−^^1^
α = 105.713 (2)°	*T* = 293 K
β = 103.367 (2)°	Block, colourless
γ = 91.051 (2)°	0.21 × 0.19 × 0.18 mm
*V* = 2674.4 (3) Å^3^	

### Data collection

**Table:**

Bruker Kappa APEXII diffractometer	11499 independent reflections
Radiation source: fine-focus sealed tube	7063 reflections with *I* > 2σ(*I*)
Graphite monochromator	*R*_int_ = 0.044
Detector resolution: 0 pixels mm^-1^	θ_max_ = 26.9°, θ_min_ = 1.6°
ω and φ scans	*h* = −16→16
Absorption correction: multi-scan (*SADABS*; Bruker, 2004)	*k* = −16→16
*T*_min_ = 0.967, *T*_max_ = 0.974	*l* = −21→21
87748 measured reflections	

### Refinement

**Table:**

Refinement on *F*^2^	Primary atom site location: structure-invariant direct methods
Least-squares matrix: full	Secondary atom site location: difference Fourier map
*R*[*F*^2^ > 2σ(*F*^2^)] = 0.058	Hydrogen site location: inferred from neighbouring sites
*wR*(*F*^2^) = 0.181	H-atom parameters constrained
*S* = 1.11	*w* = 1/[σ^2^(*F*_o_^2^) + (0.0894*P*)^2^ + 0.2555*P*] where *P* = (*F*_o_^2^ + 2*F*_c_^2^)/3
11499 reflections	(Δ/σ)_max_ < 0.001
577 parameters	Δρ_max_ = 0.38 e Å^−^^3^
2 restraints	Δρ_min_ = −0.31 e Å^−^^3^

### Special details

**Table:**

Geometry. All e.s.d.'s (except the e.s.d. in the dihedral angle between two l.s. planes) are estimated using the full covariance matrix. The cell e.s.d.'s are taken into account individually in the estimation of e.s.d.'s in distances, angles and torsion angles; correlations between e.s.d.'s in cell parameters are only used when they are defined by crystal symmetry. An approximate (isotropic) treatment of cell e.s.d.'s is used for estimating e.s.d.'s involving l.s. planes.
Refinement. Refinement of *F*^2^ against ALL reflections. The weighted *R*-factor *wR* and goodness of fit *S* are based on *F*^2^, conventional *R*-factors *R* are based on *F*, with *F* set to zero for negative *F*^2^. The threshold expression of *F*^2^ > σ(*F*^2^) is used only for calculating *R*-factors(gt) *etc*. and is not relevant to the choice of reflections for refinement. *R*-factors based on *F*^2^ are statistically about twice as large as those based on *F*, and *R*-factors based on ALL data will be even larger.

### Fractional atomic coordinates and isotropic or equivalent isotropic displacement parameters (Å^2^)

**Table:**

	*x*	*y*	*z*	*U*_iso_*/*U*_eq_	
C1A	0.73023 (17)	0.01119 (19)	0.11139 (15)	0.0368 (5)	
C1B	0.25621 (18)	0.57859 (18)	0.12320 (15)	0.0374 (5)	
C2A	0.72616 (16)	0.11182 (17)	0.17862 (14)	0.0316 (5)	
C2B	0.24268 (17)	0.51805 (17)	0.18571 (15)	0.0346 (5)	
C3A	0.61102 (16)	0.13082 (17)	0.18449 (15)	0.0338 (5)	
C3B	0.12348 (17)	0.50010 (17)	0.18260 (14)	0.0331 (5)	
C4A	0.53098 (17)	0.04635 (18)	0.14696 (16)	0.0369 (5)	
C4B	0.04671 (18)	0.55272 (17)	0.13823 (15)	0.0351 (5)	
C5A	0.64378 (18)	−0.06481 (18)	0.07503 (15)	0.0374 (5)	
C5B	0.17571 (19)	0.62288 (18)	0.07933 (15)	0.0376 (5)	
C6A	0.5327 (2)	−0.2160 (2)	0.0028 (2)	0.0587 (7)	
H1	0.5385	−0.2783	0.0232	0.070*	
H2	0.4997	−0.2371	−0.0582	0.070*	
C6B	0.0723 (2)	0.7147 (2)	−0.00442 (19)	0.0544 (7)	
H3	0.0482	0.6875	−0.0663	0.065*	
H4	0.0746	0.7911	0.0115	0.065*	
C7A	0.4711 (2)	−0.1373 (2)	0.05076 (18)	0.0514 (7)	
H5	0.4109	−0.1195	0.0119	0.062*	
H6	0.4456	−0.1640	0.0920	0.062*	
C7B	0.0004 (2)	0.6690 (2)	0.03965 (17)	0.0464 (6)	
H7	−0.0275	0.7244	0.0781	0.056*	
H8	−0.0582	0.6229	−0.0018	0.056*	
C8A	0.78204 (18)	0.20686 (19)	0.15986 (16)	0.0387 (6)	
C8B	0.2955 (2)	0.4118 (2)	0.16477 (18)	0.0431 (6)	
C9A	0.87762 (17)	0.19669 (18)	0.28932 (15)	0.0346 (5)	
C9B	0.38129 (18)	0.5008 (2)	0.30110 (17)	0.0419 (6)	
C10A	0.79719 (16)	0.11549 (17)	0.26521 (14)	0.0313 (5)	
C10B	0.30771 (17)	0.56908 (18)	0.27676 (15)	0.0352 (5)	
C11A	0.78829 (18)	0.05466 (18)	0.31873 (15)	0.0366 (5)	
H11A	0.7347	0.0003	0.3030	0.044*	
C11B	0.29907 (19)	0.66252 (19)	0.33363 (16)	0.0410 (6)	
H11B	0.2503	0.7086	0.3179	0.049*	
C12A	0.8622 (2)	0.0776 (2)	0.39682 (16)	0.0450 (6)	
C12B	0.3646 (2)	0.6868 (2)	0.41515 (17)	0.0515 (6)	
C13A	0.9416 (2)	0.1583 (2)	0.42154 (18)	0.0540 (7)	
H13A	0.9885	0.1732	0.4753	0.065*	
C13B	0.4378 (2)	0.6207 (3)	0.44003 (19)	0.0598 (7)	
H13B	0.4805	0.6392	0.4957	0.072*	
C14A	0.9517 (2)	0.2178 (2)	0.36629 (17)	0.0480 (6)	
H14A	1.0070	0.2703	0.3811	0.058*	
C14B	0.4481 (2)	0.5263 (2)	0.3822 (2)	0.0570 (7)	
H14B	0.4985	0.4815	0.3977	0.068*	
C31A	0.58488 (19)	0.2271 (2)	0.23432 (18)	0.0440 (6)	
C31B	0.08794 (18)	0.43654 (18)	0.22841 (15)	0.0378 (5)	
C32A	0.6616 (2)	0.32226 (19)	0.27750 (18)	0.0447 (6)	
C32B	0.15700 (18)	0.36977 (19)	0.27329 (16)	0.0384 (6)	
C33A	0.7186 (2)	0.3388 (2)	0.36101 (19)	0.0568 (7)	
H33A	0.7120	0.2892	0.3902	0.068*	
C33B	0.2000 (2)	0.4052 (2)	0.36081 (17)	0.0512 (7)	
H33B	0.1920	0.4737	0.3910	0.061*	
C34A	0.7868 (3)	0.4317 (3)	0.4018 (2)	0.0725 (9)	
H34A	0.8246	0.4441	0.4586	0.087*	
C34B	0.2551 (2)	0.3383 (3)	0.4037 (2)	0.0655 (8)	
H34B	0.2849	0.3622	0.4625	0.079*	
C35A	0.7976 (3)	0.5016 (2)	0.3598 (2)	0.0720 (9)	
C35B	0.2651 (3)	0.2381 (3)	0.3594 (2)	0.0705 (9)	
C36A	0.7365 (3)	0.4889 (2)	0.2770 (2)	0.0674 (9)	
H36A	0.7409	0.5407	0.2493	0.081*	
C36B	0.2220 (2)	0.2015 (2)	0.2736 (2)	0.0613 (8)	
H36B	0.2296	0.1326	0.2441	0.074*	
C37A	0.6699 (2)	0.3989 (2)	0.2368 (2)	0.0553 (7)	
H37A	0.6295	0.3893	0.1812	0.066*	
C37B	0.1672 (2)	0.2668 (2)	0.23076 (18)	0.0522 (7)	
H37B	0.1366	0.2413	0.1723	0.063*	
N1A	0.82389 (17)	−0.01200 (18)	0.09059 (15)	0.0521 (6)	
N1B	0.35638 (17)	0.59169 (17)	0.11129 (14)	0.0462 (5)	
N2A	0.43450 (16)	0.05275 (18)	0.15897 (17)	0.0598 (7)	
H9	0.3872	0.0004	0.1339	0.072*	
H10	0.4186	0.1093	0.1919	0.072*	
N2B	−0.05243 (15)	0.55052 (17)	0.14117 (14)	0.0487 (5)	
H11	−0.0962	0.5834	0.1123	0.058*	
H12	−0.0743	0.5162	0.1720	0.058*	
N3A	0.86499 (15)	0.24959 (15)	0.22690 (13)	0.0396 (5)	
H3A	0.9056	0.3037	0.2306	0.047*	
N3B	0.37179 (15)	0.41016 (16)	0.23522 (15)	0.0455 (5)	
H3B	0.4099	0.3581	0.2382	0.055*	
N4A	0.54899 (14)	−0.04556 (15)	0.09405 (13)	0.0380 (5)	
N4B	0.07354 (15)	0.60967 (15)	0.08717 (13)	0.0389 (5)	
N5A	0.63672 (17)	−0.15890 (16)	0.02111 (14)	0.0494 (5)	
H5A	0.6877	−0.1842	−0.0010	0.059*	
N5B	0.17403 (17)	0.68062 (16)	0.02601 (13)	0.0470 (5)	
H5B	0.2297	0.6965	0.0108	0.056*	
O1A	0.83315 (16)	−0.09805 (16)	0.03680 (14)	0.0713 (6)	
O1B	0.37291 (15)	0.64175 (16)	0.06023 (14)	0.0649 (6)	
O2A	0.90296 (14)	0.05404 (16)	0.12491 (13)	0.0658 (6)	
O2B	0.43195 (15)	0.55275 (17)	0.15150 (14)	0.0625 (5)	
O3A	0.75237 (14)	0.23385 (15)	0.09537 (11)	0.0525 (5)	
O3B	0.27203 (16)	0.34371 (14)	0.09631 (13)	0.0573 (5)	
O4A	0.49204 (14)	0.24063 (15)	0.24575 (16)	0.0674 (6)	
O4B	−0.00657 (13)	0.42797 (14)	0.23356 (12)	0.0522 (5)	
Cl1	0.85274 (7)	0.00066 (7)	0.46504 (5)	0.0701 (2)	
Cl2	0.88459 (12)	0.61410 (9)	0.40925 (9)	0.1355 (5)	
Cl3	0.35428 (8)	0.80620 (7)	0.48853 (5)	0.0795 (3)	
Cl4	0.33373 (11)	0.15562 (10)	0.41399 (9)	0.1317 (5)	

### Atomic displacement parameters (Å^2^)

**Table:**

	*U*^11^	*U*^22^	*U*^33^	*U*^12^	*U*^13^	*U*^23^
C1A	0.0333 (12)	0.0421 (14)	0.0393 (13)	−0.0009 (10)	0.0141 (10)	0.0147 (11)
C1B	0.0401 (13)	0.0322 (12)	0.0439 (14)	0.0005 (10)	0.0169 (11)	0.0121 (11)
C2A	0.0291 (11)	0.0337 (12)	0.0363 (12)	−0.0016 (9)	0.0094 (9)	0.0161 (10)
C2B	0.0329 (12)	0.0300 (12)	0.0437 (13)	0.0007 (9)	0.0127 (10)	0.0126 (10)
C3A	0.0276 (11)	0.0340 (12)	0.0452 (14)	0.0001 (9)	0.0082 (10)	0.0211 (11)
C3B	0.0331 (11)	0.0306 (12)	0.0387 (13)	0.0021 (9)	0.0119 (10)	0.0121 (10)
C4A	0.0266 (11)	0.0384 (13)	0.0527 (15)	0.0051 (10)	0.0086 (10)	0.0250 (12)
C4B	0.0377 (12)	0.0266 (11)	0.0428 (13)	−0.0002 (9)	0.0147 (10)	0.0092 (10)
C5A	0.0448 (14)	0.0368 (13)	0.0352 (13)	−0.0020 (10)	0.0086 (11)	0.0190 (11)
C5B	0.0462 (14)	0.0284 (12)	0.0378 (13)	−0.0081 (10)	0.0140 (11)	0.0062 (10)
C6A	0.0625 (18)	0.0456 (16)	0.0588 (18)	−0.0109 (14)	0.0047 (14)	0.0091 (14)
C6B	0.0631 (18)	0.0467 (16)	0.0551 (17)	−0.0054 (13)	0.0046 (14)	0.0262 (14)
C7A	0.0420 (14)	0.0478 (16)	0.0583 (17)	−0.0159 (12)	−0.0021 (12)	0.0185 (13)
C7B	0.0533 (15)	0.0392 (14)	0.0484 (15)	0.0080 (12)	0.0071 (12)	0.0195 (12)
C8A	0.0370 (13)	0.0404 (13)	0.0458 (15)	−0.0007 (10)	0.0135 (11)	0.0210 (12)
C8B	0.0468 (14)	0.0382 (14)	0.0528 (16)	0.0038 (11)	0.0258 (13)	0.0153 (13)
C9A	0.0287 (11)	0.0335 (12)	0.0433 (14)	0.0022 (9)	0.0117 (10)	0.0111 (10)
C9B	0.0337 (12)	0.0458 (15)	0.0560 (16)	0.0050 (11)	0.0159 (12)	0.0264 (13)
C10A	0.0272 (11)	0.0329 (12)	0.0373 (12)	0.0051 (9)	0.0106 (9)	0.0135 (10)
C10B	0.0304 (11)	0.0336 (12)	0.0472 (14)	0.0001 (9)	0.0122 (10)	0.0185 (11)
C11A	0.0354 (12)	0.0375 (13)	0.0398 (13)	0.0017 (10)	0.0122 (10)	0.0133 (11)
C11B	0.0403 (13)	0.0382 (14)	0.0480 (15)	0.0041 (11)	0.0134 (12)	0.0158 (12)
C12A	0.0464 (14)	0.0518 (16)	0.0438 (15)	0.0127 (12)	0.0123 (12)	0.0234 (12)
C12B	0.0568 (16)	0.0493 (13)	0.0458 (16)	−0.0084 (13)	0.0116 (13)	0.0109 (10)
C13A	0.0505 (16)	0.0576 (17)	0.0457 (16)	0.0044 (13)	−0.0027 (13)	0.0135 (14)
C13B	0.0502 (16)	0.0732 (17)	0.0550 (18)	−0.0099 (15)	−0.0002 (13)	0.0286 (13)
C14A	0.0411 (14)	0.0469 (15)	0.0494 (16)	−0.0024 (12)	0.0022 (12)	0.0108 (13)
C14B	0.0417 (14)	0.0625 (15)	0.076 (2)	0.0026 (13)	0.0063 (14)	0.0413 (13)
C31A	0.0343 (13)	0.0439 (15)	0.0621 (17)	0.0059 (11)	0.0152 (12)	0.0258 (13)
C31B	0.0384 (13)	0.0347 (13)	0.0426 (14)	−0.0016 (10)	0.0127 (11)	0.0125 (11)
C32A	0.0450 (14)	0.0359 (14)	0.0596 (17)	0.0096 (11)	0.0250 (13)	0.0134 (12)
C32B	0.0352 (12)	0.0405 (14)	0.0468 (15)	−0.0007 (10)	0.0130 (11)	0.0222 (12)
C33A	0.0646 (18)	0.0522 (17)	0.0576 (19)	0.0059 (14)	0.0272 (15)	0.0118 (14)
C33B	0.0536 (16)	0.0526 (17)	0.0474 (16)	−0.0011 (13)	0.0112 (13)	0.0157 (13)
C34A	0.086 (2)	0.068 (2)	0.0472 (18)	−0.0129 (18)	0.0137 (16)	−0.0079 (16)
C34B	0.0640 (19)	0.071 (2)	0.0616 (19)	−0.0087 (16)	−0.0005 (15)	0.0338 (17)
C35A	0.081 (2)	0.0463 (18)	0.081 (2)	−0.0130 (16)	0.033 (2)	−0.0047 (17)
C35B	0.065 (2)	0.066 (2)	0.085 (3)	0.0012 (16)	−0.0011 (18)	0.046 (2)
C36A	0.081 (2)	0.0403 (16)	0.083 (2)	−0.0071 (15)	0.0272 (19)	0.0157 (16)
C36B	0.0632 (18)	0.0380 (15)	0.085 (2)	0.0083 (14)	0.0178 (17)	0.0209 (16)
C37A	0.0612 (17)	0.0410 (15)	0.0678 (19)	0.0065 (13)	0.0170 (15)	0.0209 (14)
C37B	0.0610 (17)	0.0420 (15)	0.0532 (17)	−0.0061 (13)	0.0111 (13)	0.0164 (13)
N1A	0.0467 (13)	0.0525 (14)	0.0562 (14)	−0.0067 (11)	0.0252 (11)	0.0036 (12)
N1B	0.0464 (12)	0.0459 (12)	0.0585 (14)	0.0024 (10)	0.0261 (11)	0.0238 (11)
N2A	0.0328 (11)	0.0460 (13)	0.102 (2)	0.0006 (10)	0.0170 (12)	0.0228 (13)
N2B	0.0347 (11)	0.0559 (14)	0.0679 (15)	0.0057 (10)	0.0138 (10)	0.0365 (12)
N3A	0.0358 (11)	0.0369 (11)	0.0478 (12)	−0.0079 (9)	0.0113 (9)	0.0148 (9)
N3B	0.0367 (11)	0.0390 (12)	0.0701 (15)	0.0128 (9)	0.0203 (11)	0.0242 (11)
N4A	0.0333 (10)	0.0332 (11)	0.0465 (12)	−0.0054 (8)	0.0038 (9)	0.0150 (9)
N4B	0.0395 (11)	0.0374 (11)	0.0458 (12)	0.0034 (9)	0.0114 (9)	0.0207 (9)
N5A	0.0550 (13)	0.0415 (13)	0.0496 (13)	−0.0063 (10)	0.0164 (11)	0.0072 (11)
N5B	0.0540 (13)	0.0450 (12)	0.0484 (13)	−0.0026 (10)	0.0123 (10)	0.0245 (10)
O1A	0.0693 (13)	0.0580 (13)	0.0798 (15)	−0.0087 (10)	0.0425 (12)	−0.0111 (11)
O1B	0.0609 (12)	0.0743 (14)	0.0861 (15)	0.0016 (10)	0.0382 (11)	0.0497 (12)
O2A	0.0440 (11)	0.0638 (13)	0.0797 (15)	−0.0131 (10)	0.0311 (10)	−0.0082 (11)
O2B	0.0429 (10)	0.0775 (14)	0.0871 (15)	0.0110 (10)	0.0309 (10)	0.0434 (12)
O3A	0.0560 (11)	0.0606 (12)	0.0487 (11)	−0.0107 (9)	0.0099 (9)	0.0320 (10)
O3B	0.0764 (13)	0.0379 (10)	0.0635 (13)	0.0091 (9)	0.0317 (11)	0.0116 (10)
O4A	0.0416 (10)	0.0435 (11)	0.1263 (19)	0.0142 (8)	0.0356 (12)	0.0259 (12)
O4B	0.0394 (10)	0.0571 (11)	0.0744 (13)	0.0009 (8)	0.0194 (9)	0.0379 (10)
Cl1	0.0825 (5)	0.0836 (6)	0.0548 (5)	0.0085 (4)	0.0105 (4)	0.0422 (4)
Cl2	0.1648 (12)	0.0872 (8)	0.1189 (10)	−0.0690 (8)	0.0301 (8)	−0.0201 (7)
Cl3	0.1047 (7)	0.0664 (5)	0.0559 (5)	−0.0044 (5)	0.0201 (4)	−0.0011 (4)
Cl4	0.1366 (10)	0.1045 (9)	0.1475 (11)	0.0217 (7)	−0.0301 (8)	0.0787 (8)

### Geometric parameters (Å, º)

**Table:**

C1A—N1A	1.358 (3)	C11A—H11A	0.9300
C1A—C5A	1.396 (3)	C11B—C12B	1.379 (4)
C1A—C2A	1.501 (3)	C11B—H11B	0.9300
C1B—C5B	1.368 (3)	C12A—C13A	1.376 (4)
C1B—N1B	1.375 (3)	C12A—Cl1	1.743 (3)
C1B—C2B	1.515 (3)	C12B—C13B	1.372 (4)
C2A—C10A	1.514 (3)	C12B—Cl3	1.745 (3)
C2A—C3A	1.541 (3)	C13A—C14A	1.390 (4)
C2A—C8A	1.577 (3)	C13A—H13A	0.9300
C2B—C10B	1.521 (3)	C13B—C14B	1.385 (4)
C2B—C3B	1.548 (3)	C13B—H13B	0.9300
C2B—C8B	1.563 (3)	C14A—H14A	0.9300
C3A—C4A	1.412 (3)	C14B—H14B	0.9300
C3A—C31A	1.414 (3)	C31A—O4A	1.271 (3)
C3B—C4B	1.410 (3)	C31A—C32A	1.491 (4)
C3B—C31B	1.415 (3)	C31B—O4B	1.255 (3)
C4A—N2A	1.314 (3)	C31B—C32B	1.490 (3)
C4A—N4A	1.355 (3)	C32A—C33A	1.376 (4)
C4B—N2B	1.299 (3)	C32A—C37A	1.379 (4)
C4B—N4B	1.375 (3)	C32B—C37B	1.378 (4)
C5A—N5A	1.310 (3)	C32B—C33B	1.383 (3)
C5A—N4A	1.352 (3)	C33A—C34A	1.408 (4)
C5B—N5B	1.318 (3)	C33A—H33A	0.9300
C5B—N4B	1.375 (3)	C33B—C34B	1.391 (4)
C6A—N5A	1.460 (3)	C33B—H33B	0.9300
C6A—C7A	1.497 (4)	C34A—C35A	1.326 (5)
C6A—H1	0.9700	C34A—H34A	0.9300
C6A—H2	0.9700	C34B—C35B	1.355 (5)
C6B—N5B	1.428 (3)	C34B—H34B	0.9300
C6B—C7B	1.522 (4)	C35A—C36A	1.392 (5)
C6B—H3	0.9700	C35A—Cl2	1.734 (3)
C6B—H4	0.9700	C35B—C36B	1.359 (4)
C7A—N4A	1.469 (3)	C35B—Cl4	1.736 (3)
C7A—H5	0.9700	C36A—C37A	1.369 (4)
C7A—H6	0.9700	C36A—H36A	0.9300
C7B—N4B	1.470 (3)	C36B—C37B	1.373 (4)
C7B—H7	0.9700	C36B—H36B	0.9300
C7B—H8	0.9700	C37A—H37A	0.9300
C8A—O3A	1.208 (3)	C37B—H37B	0.9300
C8A—N3A	1.346 (3)	N1A—O2A	1.252 (3)
C8B—O3B	1.218 (3)	N1A—O1A	1.271 (3)
C8B—N3B	1.358 (3)	N1B—O2B	1.257 (3)
C9A—C14A	1.372 (3)	N1B—O1B	1.264 (3)
C9A—N3A	1.387 (3)	N2A—H9	0.8600
C9A—C10A	1.390 (3)	N2A—H10	0.8600
C9B—N3B	1.371 (3)	N2B—H11	0.8600
C9B—C14B	1.380 (4)	N2B—H12	0.8600
C9B—C10B	1.394 (3)	N3A—H3A	0.8600
C10A—C11A	1.376 (3)	N3B—H3B	0.8600
C10B—C11B	1.363 (3)	N5A—H5A	0.8600
C11A—C12A	1.383 (3)	N5B—H5B	0.8600
			
N1A—C1A—C5A	118.1 (2)	C13B—C12B—C11B	122.2 (3)
N1A—C1A—C2A	119.2 (2)	C13B—C12B—Cl3	119.2 (2)
C5A—C1A—C2A	122.4 (2)	C11B—C12B—Cl3	118.6 (2)
C5B—C1B—N1B	118.0 (2)	C12A—C13A—C14A	120.1 (2)
C5B—C1B—C2B	124.2 (2)	C12A—C13A—H13A	119.9
N1B—C1B—C2B	117.7 (2)	C14A—C13A—H13A	119.9
C1A—C2A—C10A	112.31 (18)	C12B—C13B—C14B	120.0 (3)
C1A—C2A—C3A	111.22 (18)	C12B—C13B—H13B	120.0
C10A—C2A—C3A	111.30 (17)	C14B—C13B—H13B	120.0
C1A—C2A—C8A	109.75 (17)	C9A—C14A—C13A	118.0 (2)
C10A—C2A—C8A	99.90 (17)	C9A—C14A—H14A	121.0
C3A—C2A—C8A	111.88 (18)	C13A—C14A—H14A	121.0
C1B—C2B—C10B	113.39 (18)	C9B—C14B—C13B	118.0 (3)
C1B—C2B—C3B	110.24 (18)	C9B—C14B—H14B	121.0
C10B—C2B—C3B	111.44 (18)	C13B—C14B—H14B	121.0
C1B—C2B—C8B	109.31 (18)	O4A—C31A—C3A	122.5 (2)
C10B—C2B—C8B	100.11 (19)	O4A—C31A—C32A	113.5 (2)
C3B—C2B—C8B	112.00 (18)	C3A—C31A—C32A	124.0 (2)
C4A—C3A—C31A	118.5 (2)	O4B—C31B—C3B	123.4 (2)
C4A—C3A—C2A	119.3 (2)	O4B—C31B—C32B	112.48 (19)
C31A—C3A—C2A	121.84 (19)	C3B—C31B—C32B	124.1 (2)
C4B—C3B—C31B	117.4 (2)	C33A—C32A—C37A	119.1 (3)
C4B—C3B—C2B	120.74 (19)	C33A—C32A—C31A	120.3 (2)
C31B—C3B—C2B	121.8 (2)	C37A—C32A—C31A	120.4 (3)
N2A—C4A—N4A	116.1 (2)	C37B—C32B—C33B	118.6 (2)
N2A—C4A—C3A	122.5 (2)	C37B—C32B—C31B	120.4 (2)
N4A—C4A—C3A	121.37 (19)	C33B—C32B—C31B	120.3 (2)
N2B—C4B—N4B	115.5 (2)	C32A—C33A—C34A	119.3 (3)
N2B—C4B—C3B	123.7 (2)	C32A—C33A—H33A	120.3
N4B—C4B—C3B	120.8 (2)	C34A—C33A—H33A	120.3
N5A—C5A—N4A	109.7 (2)	C32B—C33B—C34B	120.1 (3)
N5A—C5A—C1A	130.0 (2)	C32B—C33B—H33B	120.0
N4A—C5A—C1A	120.3 (2)	C34B—C33B—H33B	120.0
N5B—C5B—C1B	132.2 (2)	C35A—C34A—C33A	120.4 (3)
N5B—C5B—N4B	107.5 (2)	C35A—C34A—H34A	119.8
C1B—C5B—N4B	120.3 (2)	C33A—C34A—H34A	119.8
N5A—C6A—C7A	103.5 (2)	C35B—C34B—C33B	119.5 (3)
N5A—C6A—H1	111.1	C35B—C34B—H34B	120.2
C7A—C6A—H1	111.1	C33B—C34B—H34B	120.2
N5A—C6A—H2	111.1	C34A—C35A—C36A	121.1 (3)
C7A—C6A—H2	111.1	C34A—C35A—Cl2	120.3 (3)
H1—C6A—H2	109.0	C36A—C35A—Cl2	118.6 (3)
N5B—C6B—C7B	103.9 (2)	C34B—C35B—C36B	121.2 (3)
N5B—C6B—H3	111.0	C34B—C35B—Cl4	118.9 (3)
C7B—C6B—H3	111.0	C36B—C35B—Cl4	119.8 (3)
N5B—C6B—H4	111.0	C37A—C36A—C35A	118.8 (3)
C7B—C6B—H4	111.0	C37A—C36A—H36A	120.6
H3—C6B—H4	109.0	C35A—C36A—H36A	120.6
N4A—C7A—C6A	103.4 (2)	C35B—C36B—C37B	119.7 (3)
N4A—C7A—H5	111.1	C35B—C36B—H36B	120.2
C6A—C7A—H5	111.1	C37B—C36B—H36B	120.2
N4A—C7A—H6	111.1	C36A—C37A—C32A	121.2 (3)
C6A—C7A—H6	111.1	C36A—C37A—H37A	119.4
H5—C7A—H6	109.1	C32A—C37A—H37A	119.4
N4B—C7B—C6B	102.0 (2)	C36B—C37B—C32B	120.9 (3)
N4B—C7B—H7	111.4	C36B—C37B—H37B	119.6
C6B—C7B—H7	111.4	C32B—C37B—H37B	119.6
N4B—C7B—H8	111.4	O2A—N1A—O1A	119.3 (2)
C6B—C7B—H8	111.4	O2A—N1A—C1A	118.8 (2)
H7—C7B—H8	109.2	O1A—N1A—C1A	122.0 (2)
O3A—C8A—N3A	128.0 (2)	O2B—N1B—O1B	119.92 (19)
O3A—C8A—C2A	123.8 (2)	O2B—N1B—C1B	119.3 (2)
N3A—C8A—C2A	108.21 (19)	O1B—N1B—C1B	120.8 (2)
O3B—C8B—N3B	126.7 (2)	C4A—N2A—H9	120.0
O3B—C8B—C2B	125.1 (2)	C4A—N2A—H10	120.0
N3B—C8B—C2B	108.2 (2)	H9—N2A—H10	120.0
C14A—C9A—N3A	129.1 (2)	C4B—N2B—H11	120.0
C14A—C9A—C10A	121.4 (2)	C4B—N2B—H12	120.0
N3A—C9A—C10A	109.4 (2)	H11—N2B—H12	120.0
N3B—C9B—C14B	128.8 (2)	C8A—N3A—C9A	112.49 (18)
N3B—C9B—C10B	109.9 (2)	C8A—N3A—H3A	123.8
C14B—C9B—C10B	121.2 (3)	C9A—N3A—H3A	123.8
C11A—C10A—C9A	120.9 (2)	C8B—N3B—C9B	112.4 (2)
C11A—C10A—C2A	129.1 (2)	C8B—N3B—H3B	123.8
C9A—C10A—C2A	109.95 (18)	C9B—N3B—H3B	123.8
C11B—C10B—C9B	120.4 (2)	C5A—N4A—C4A	123.45 (19)
C11B—C10B—C2B	130.2 (2)	C5A—N4A—C7A	110.8 (2)
C9B—C10B—C2B	109.3 (2)	C4A—N4A—C7A	125.7 (2)
C10A—C11A—C12A	117.4 (2)	C5B—N4B—C4B	122.8 (2)
C10A—C11A—H11A	121.3	C5B—N4B—C7B	111.89 (19)
C12A—C11A—H11A	121.3	C4B—N4B—C7B	125.18 (19)
C10B—C11B—C12B	118.2 (2)	C5A—N5A—C6A	112.5 (2)
C10B—C11B—H11B	120.9	C5A—N5A—H5A	123.8
C12B—C11B—H11B	120.9	C6A—N5A—H5A	123.8
C13A—C12A—C11A	122.2 (2)	C5B—N5B—C6B	114.6 (2)
C13A—C12A—Cl1	119.8 (2)	C5B—N5B—H5B	122.7
C11A—C12A—Cl1	118.0 (2)	C6B—N5B—H5B	122.7
			
N1A—C1A—C2A—C10A	−62.0 (3)	Cl3—C12B—C13B—C14B	−179.1 (2)
C5A—C1A—C2A—C10A	111.6 (2)	N3A—C9A—C14A—C13A	174.2 (2)
N1A—C1A—C2A—C3A	172.5 (2)	C10A—C9A—C14A—C13A	−2.7 (4)
C5A—C1A—C2A—C3A	−13.9 (3)	C12A—C13A—C14A—C9A	3.1 (4)
N1A—C1A—C2A—C8A	48.2 (3)	N3B—C9B—C14B—C13B	−175.6 (2)
C5A—C1A—C2A—C8A	−138.2 (2)	C10B—C9B—C14B—C13B	2.0 (4)
C5B—C1B—C2B—C10B	−117.6 (2)	C12B—C13B—C14B—C9B	−1.6 (4)
N1B—C1B—C2B—C10B	60.9 (3)	C4A—C3A—C31A—O4A	−0.7 (4)
C5B—C1B—C2B—C3B	8.1 (3)	C2A—C3A—C31A—O4A	−173.7 (2)
N1B—C1B—C2B—C3B	−173.3 (2)	C4A—C3A—C31A—C32A	179.9 (2)
C5B—C1B—C2B—C8B	131.6 (2)	C2A—C3A—C31A—C32A	6.9 (4)
N1B—C1B—C2B—C8B	−49.8 (3)	C4B—C3B—C31B—O4B	−2.5 (4)
C1A—C2A—C3A—C4A	16.1 (3)	C2B—C3B—C31B—O4B	173.4 (2)
C10A—C2A—C3A—C4A	−110.0 (2)	C4B—C3B—C31B—C32B	175.4 (2)
C8A—C2A—C3A—C4A	139.2 (2)	C2B—C3B—C31B—C32B	−8.8 (4)
C1A—C2A—C3A—C31A	−171.0 (2)	O4A—C31A—C32A—C33A	88.0 (3)
C10A—C2A—C3A—C31A	63.0 (3)	C3A—C31A—C32A—C33A	−92.6 (3)
C8A—C2A—C3A—C31A	−47.8 (3)	O4A—C31A—C32A—C37A	−86.6 (3)
C1B—C2B—C3B—C4B	−10.7 (3)	C3A—C31A—C32A—C37A	92.8 (3)
C10B—C2B—C3B—C4B	116.1 (2)	O4B—C31B—C32B—C37B	88.3 (3)
C8B—C2B—C3B—C4B	−132.7 (2)	C3B—C31B—C32B—C37B	−89.7 (3)
C1B—C2B—C3B—C31B	173.6 (2)	O4B—C31B—C32B—C33B	−82.8 (3)
C10B—C2B—C3B—C31B	−59.6 (3)	C3B—C31B—C32B—C33B	99.1 (3)
C8B—C2B—C3B—C31B	51.7 (3)	C37A—C32A—C33A—C34A	−2.3 (4)
C31A—C3A—C4A—N2A	−2.4 (3)	C31A—C32A—C33A—C34A	−177.0 (3)
C2A—C3A—C4A—N2A	170.8 (2)	C37B—C32B—C33B—C34B	2.0 (4)
C31A—C3A—C4A—N4A	175.7 (2)	C31B—C32B—C33B—C34B	173.3 (2)
C2A—C3A—C4A—N4A	−11.1 (3)	C32A—C33A—C34A—C35A	−1.2 (5)
C31B—C3B—C4B—N2B	3.9 (3)	C32B—C33B—C34B—C35B	−0.9 (4)
C2B—C3B—C4B—N2B	−171.9 (2)	C33A—C34A—C35A—C36A	4.6 (5)
C31B—C3B—C4B—N4B	−175.2 (2)	C33A—C34A—C35A—Cl2	−178.1 (2)
C2B—C3B—C4B—N4B	8.9 (3)	C33B—C34B—C35B—C36B	−0.1 (5)
N1A—C1A—C5A—N5A	−0.7 (4)	C33B—C34B—C35B—Cl4	−179.8 (2)
C2A—C1A—C5A—N5A	−174.4 (2)	C34A—C35A—C36A—C37A	−4.5 (5)
N1A—C1A—C5A—N4A	179.7 (2)	Cl2—C35A—C36A—C37A	178.2 (2)
C2A—C1A—C5A—N4A	6.0 (3)	C34B—C35B—C36B—C37B	0.0 (5)
N1B—C1B—C5B—N5B	−1.5 (4)	Cl4—C35B—C36B—C37B	179.6 (2)
C2B—C1B—C5B—N5B	177.0 (2)	C35A—C36A—C37A—C32A	0.9 (4)
N1B—C1B—C5B—N4B	178.2 (2)	C33A—C32A—C37A—C36A	2.4 (4)
C2B—C1B—C5B—N4B	−3.2 (4)	C31A—C32A—C37A—C36A	177.1 (3)
N5A—C6A—C7A—N4A	−2.3 (3)	C35B—C36B—C37B—C32B	1.2 (4)
N5B—C6B—C7B—N4B	−2.6 (3)	C33B—C32B—C37B—C36B	−2.2 (4)
C1A—C2A—C8A—O3A	61.2 (3)	C31B—C32B—C37B—C36B	−173.5 (2)
C10A—C2A—C8A—O3A	179.4 (2)	C5A—C1A—N1A—O2A	−177.7 (2)
C3A—C2A—C8A—O3A	−62.7 (3)	C2A—C1A—N1A—O2A	−3.8 (4)
C1A—C2A—C8A—N3A	−118.9 (2)	C5A—C1A—N1A—O1A	3.1 (4)
C10A—C2A—C8A—N3A	−0.7 (2)	C2A—C1A—N1A—O1A	177.0 (2)
C3A—C2A—C8A—N3A	117.2 (2)	C5B—C1B—N1B—O2B	179.3 (2)
C1B—C2B—C8B—O3B	−58.4 (3)	C2B—C1B—N1B—O2B	0.6 (3)
C10B—C2B—C8B—O3B	−177.7 (2)	C5B—C1B—N1B—O1B	−1.1 (3)
C3B—C2B—C8B—O3B	64.1 (3)	C2B—C1B—N1B—O1B	−179.8 (2)
C1B—C2B—C8B—N3B	120.9 (2)	O3A—C8A—N3A—C9A	−178.5 (2)
C10B—C2B—C8B—N3B	1.5 (2)	C2A—C8A—N3A—C9A	1.7 (3)
C3B—C2B—C8B—N3B	−116.6 (2)	C14A—C9A—N3A—C8A	−179.1 (2)
C14A—C9A—C10A—C11A	1.1 (3)	C10A—C9A—N3A—C8A	−2.0 (3)
N3A—C9A—C10A—C11A	−176.27 (19)	O3B—C8B—N3B—C9B	177.7 (2)
C14A—C9A—C10A—C2A	178.8 (2)	C2B—C8B—N3B—C9B	−1.5 (3)
N3A—C9A—C10A—C2A	1.4 (2)	C14B—C9B—N3B—C8B	178.6 (2)
C1A—C2A—C10A—C11A	−66.7 (3)	C10B—C9B—N3B—C8B	0.8 (3)
C3A—C2A—C10A—C11A	58.7 (3)	N5A—C5A—N4A—C4A	−178.6 (2)
C8A—C2A—C10A—C11A	177.0 (2)	C1A—C5A—N4A—C4A	1.0 (3)
C1A—C2A—C10A—C9A	115.8 (2)	N5A—C5A—N4A—C7A	2.0 (3)
C3A—C2A—C10A—C9A	−118.7 (2)	C1A—C5A—N4A—C7A	−178.3 (2)
C8A—C2A—C10A—C9A	−0.4 (2)	N2A—C4A—N4A—C5A	−180.0 (2)
N3B—C9B—C10B—C11B	176.9 (2)	C3A—C4A—N4A—C5A	1.8 (3)
C14B—C9B—C10B—C11B	−1.1 (3)	N2A—C4A—N4A—C7A	−0.8 (3)
N3B—C9B—C10B—C2B	0.3 (2)	C3A—C4A—N4A—C7A	−179.0 (2)
C14B—C9B—C10B—C2B	−177.7 (2)	C6A—C7A—N4A—C5A	0.4 (3)
C1B—C2B—C10B—C11B	66.5 (3)	C6A—C7A—N4A—C4A	−178.9 (2)
C3B—C2B—C10B—C11B	−58.6 (3)	N5B—C5B—N4B—C4B	179.9 (2)
C8B—C2B—C10B—C11B	−177.2 (2)	C1B—C5B—N4B—C4B	0.0 (3)
C1B—C2B—C10B—C9B	−117.4 (2)	N5B—C5B—N4B—C7B	−4.0 (3)
C3B—C2B—C10B—C9B	117.5 (2)	C1B—C5B—N4B—C7B	176.2 (2)
C8B—C2B—C10B—C9B	−1.1 (2)	N2B—C4B—N4B—C5B	177.7 (2)
C9A—C10A—C11A—C12A	0.0 (3)	C3B—C4B—N4B—C5B	−3.1 (3)
C2A—C10A—C11A—C12A	−177.2 (2)	N2B—C4B—N4B—C7B	2.1 (3)
C9B—C10B—C11B—C12B	−0.1 (3)	C3B—C4B—N4B—C7B	−178.7 (2)
C2B—C10B—C11B—C12B	175.6 (2)	C6B—C7B—N4B—C5B	4.0 (3)
C10A—C11A—C12A—C13A	0.6 (4)	C6B—C7B—N4B—C4B	−179.9 (2)
C10A—C11A—C12A—Cl1	−179.50 (17)	N4A—C5A—N5A—C6A	−3.8 (3)
C10B—C11B—C12B—C13B	0.5 (4)	C1A—C5A—N5A—C6A	176.6 (2)
C10B—C11B—C12B—Cl3	180.00 (18)	C7A—C6A—N5A—C5A	3.8 (3)
C11A—C12A—C13A—C14A	−2.2 (4)	C1B—C5B—N5B—C6B	−178.1 (3)
Cl1—C12A—C13A—C14A	177.9 (2)	N4B—C5B—N5B—C6B	2.2 (3)
C11B—C12B—C13B—C14B	0.5 (4)	C7B—C6B—N5B—C5B	0.4 (3)

### Hydrogen-bond geometry (Å, º)

Cg1 and Cg2 are the centroids of rings C1A–C5A/N4A and C1B–C5B/N4B, respectively.

**Table:**

*D*—H···*A*	*D*—H	H···*A*	*D*···*A*	*D*—H···*A*
N3*B*—H3*B*···O4*A*	0.86	1.91	2.774 (3)	179
N5*A*—H5*A*···O1*A*	0.86	2.06	2.592 (3)	119
N5*B*—H5*B*···O1*B*	0.86	2.08	2.604 (3)	119
N2*A*—H10···O4*A*	0.86	1.85	2.510 (3)	132
N2*B*—H12···O4*B*	0.86	1.86	2.515 (3)	132
N3*A*—H3*A*···O4*B*^i^	0.86	1.96	2.823 (2)	177
N5*A*—H5*A*···O3*B*^ii^	0.86	2.42	3.131 (3)	140
N5*B*—H5*B*···O3*A*^iii^	0.86	2.26	2.899 (3)	131
C7*A*—H5···O3*A*^ii^	0.97	2.58	3.302 (3)	132
C7*A*—H6···O1*B*^iv^	0.97	2.59	3.221 (3)	123
C7*A*—H5···*Cg*1^ii^	0.97	2.89	3.679 (3)	139
C7*B*—H8···*Cg*2^v^	0.97	2.85	3.747 (3)	155

Symmetry codes: (i) *x*+1, *y*, *z*; (ii) −*x*+1, −*y*, −*z*; (iii) −*x*+1, −*y*+1, −*z*; (iv) *x*, *y*−1, *z*; (v) −*x*, −*y*+1, −*z*.
