# Supplementary figures and images for: Crystal structure of 5-amino-5′-chloro-6-(4-chloro­benzo­yl)-8-nitro-2,3-di­hydro-1H-spiro­[imidazo[1,2-a]pyridine-7,3′-indolin]-2′-one including an unknown solvent mol­ecule
Source: Acta Crystallogr Sect E Struct Rep Online. 2014 Aug 6;70(Pt 9):o971–2. doi: 10.1107/S1600536814017486 (PMC4186207; doi:10.1107/S1600536814017486)

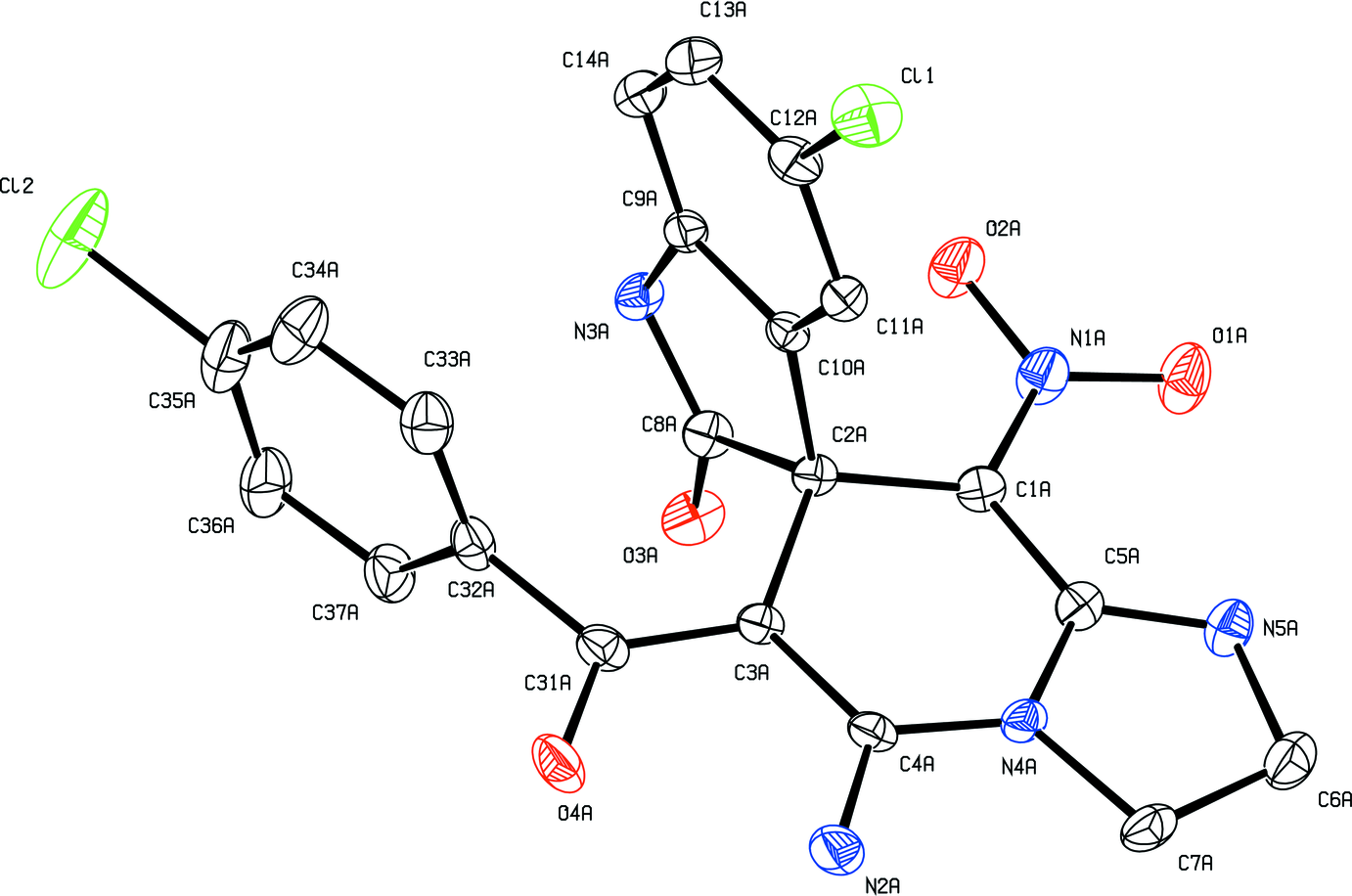

Supplement: Supplementary file 4 [file e-70-0o971-fig1.tif]

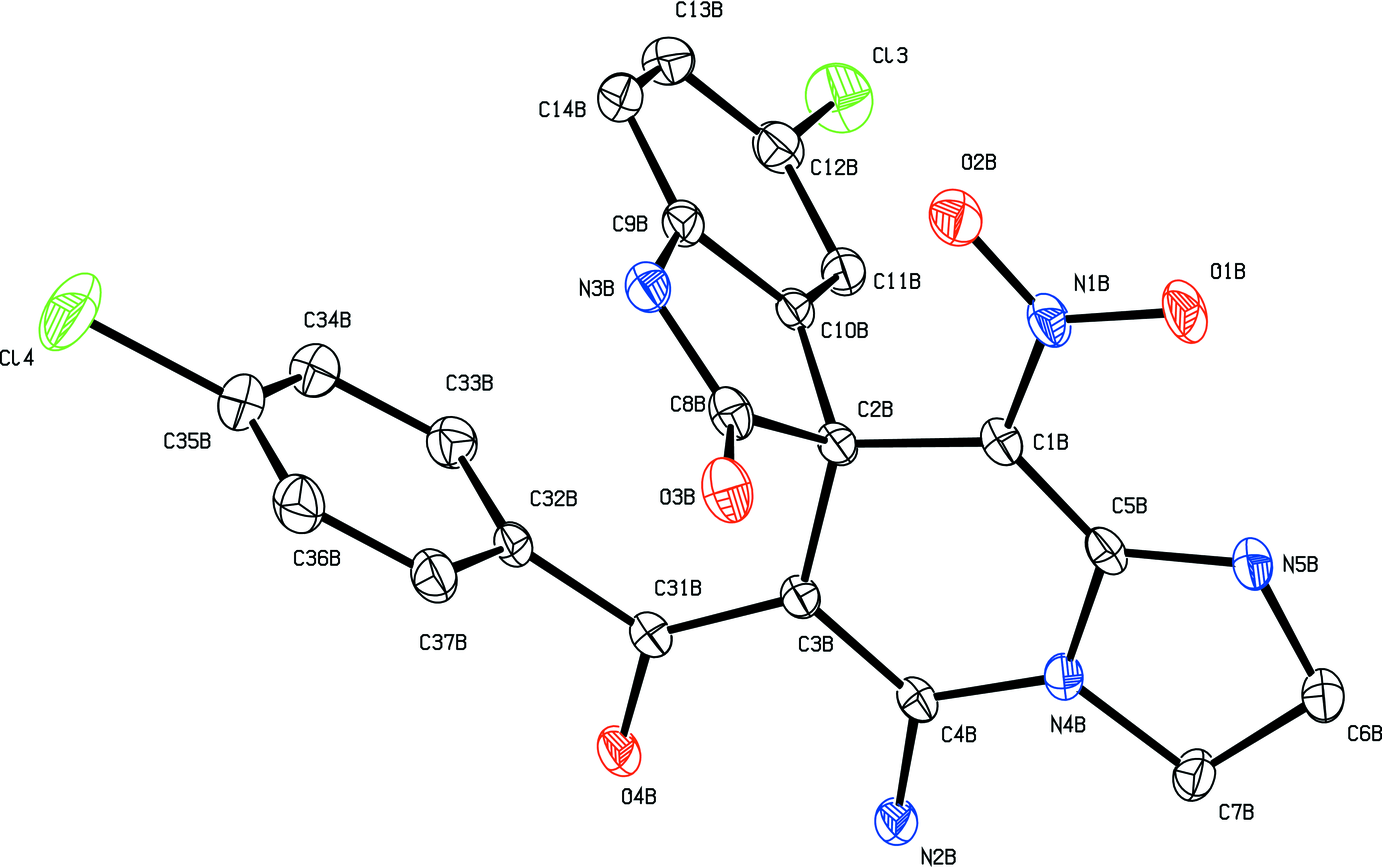

Supplement: Supplementary file 5 [file e-70-0o971-fig2.tif]

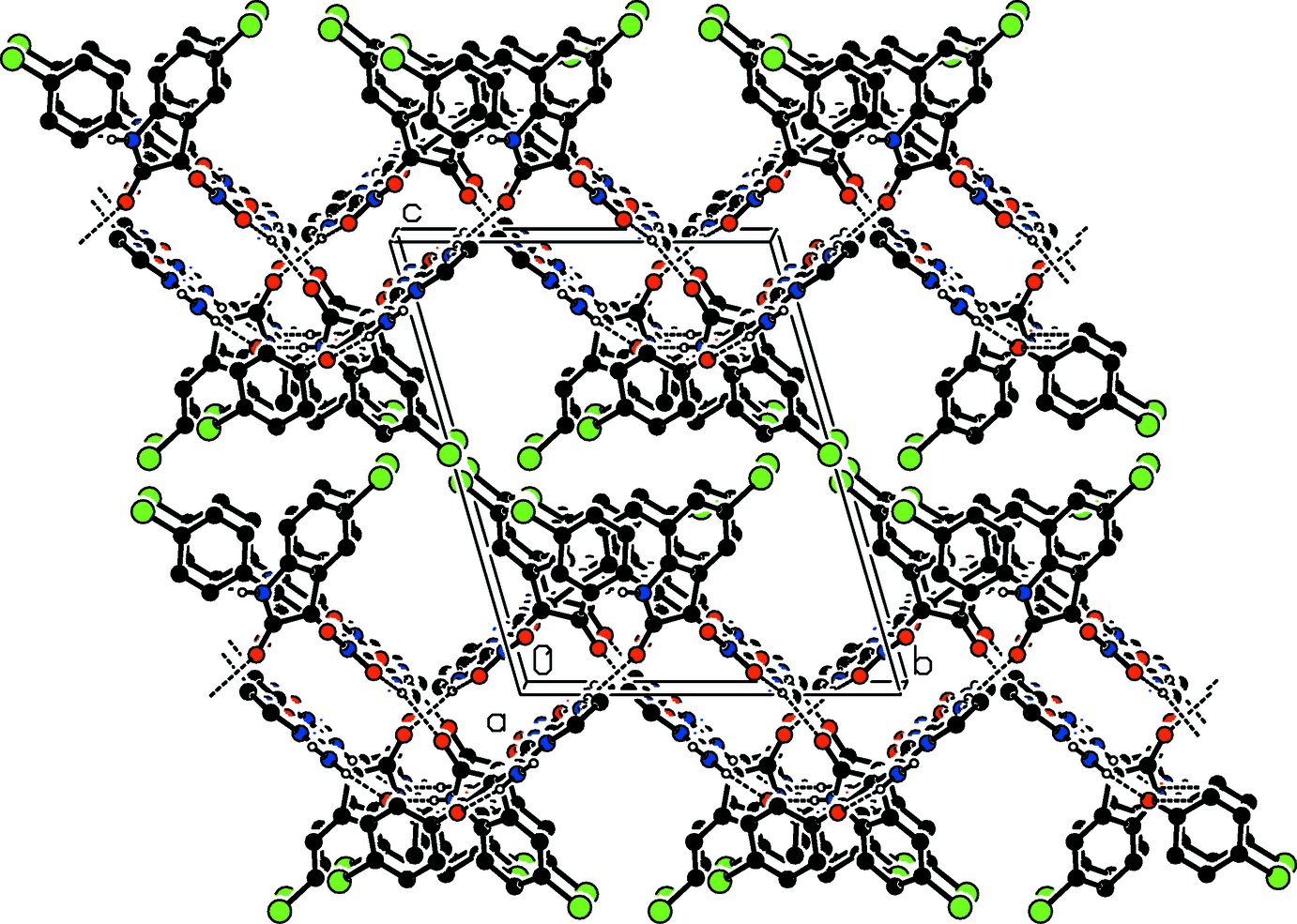

Supplement: Supplementary file 6 [file e-70-0o971-fig3.tif]
